# Use of ChatGPT for Urinary Symptom Management Among People With Spinal Cord Injury or Disease: Qualitative Study

**DOI:** 10.2196/70339

**Published:** 2025-05-29

**Authors:** Bat-Zion Hose, Amanda K Rounds, Ishaan Nandwani, Deanna-Nicole Busog, Traber Davis Giardina, Helen Haskell, Kelly M Smith, Kristen E Miller

**Affiliations:** 1 MedStar Health National Center for Human Factors in Healthcare, 3007 Tilden St NW, Suite 6N, Washington, DC, 20008, United States, 1 608-719-9991; 2 Georgetown University School of Medicine, 3900 Reservoir Rd NW, Washington, DC, 20007, United States, 1 202-687-0100; 3 MedStar National Rehabilitation Hospital, Washington, DC, United States; 4 Center for Innovations in Quality, Effectiveness and Safety, Michael E. DeBakey VA Medical Center and Baylor College of Medicine, Houston, TX, United States; 5 Department of Medicine, Baylor College of Medicine, Houston, TX, United States; 6 Mothers Against Medical Error, Columbia, SC, United States; 7 Michael Garron Hospital, Toronto, ON, Canada; 8 Institute of Health Policy, Management, and Evaluation, The University of Toronto, Toronto, ON, Canada

**Keywords:** ChatGPT, urinary symptom management, spinal cord injury, trust in artificial intelligence

## Abstract

**Background:**

Individuals with spinal cord injury or disease (SCI/D) experience disproportionately high rates of recurrent urinary tract infections, which are often complicated by atypical symptoms and delayed diagnoses. Patient-centered tools, like the Urinary Symptom Questionnaires for Neurogenic Bladder (USQNB), have been developed to support symptom assessment yet remain underused. Generative artificial intelligence tools such as ChatGPT may offer a more usable approach to improving symptom management by providing real-time, tailored health information directly to patients.

**Objective:**

This study explores the role of ChatGPT (version 3.5) in supporting urinary symptom management for individuals with SCI/D, focusing on its perceived accuracy, usefulness, and impact on health care engagement and self-management practices.

**Methods:**

A total of 30 individuals with SCI/D were recruited through advocacy groups and health care networks. Using realistic, scenario-based testing derived from validated tools for symptom management with SCI/D, such as the USQNB, participants interacted with ChatGPT to seek advice for urinary symptoms. Follow-up interviews were conducted remotely to assess individuals’ experiences using ChatGPT for urinary symptom management. Data were analyzed using inductive content analysis, with themes refined iteratively through a consensus-based process.

**Results:**

People with SCI/D reported high levels of trust in ChatGPT’s recommendations, with all 30 participants agreeing or strongly agreeing with the advice provided. ChatGPT’s responses were perceived as clear and comparable to professional medical advice. Participants mentioned concerns about the lack of sources and integration with patient-specific data. ChatGPT influenced individuals’ decision-making by supporting symptom assessment and guiding participants on when to seek professional care or pursue self-management strategies.

**Conclusions:**

ChatGPT is a promising tool for symptom assessment and managing chronic conditions such as urinary symptoms in individuals with SCI/D. While ChatGPT enhances accessibility to health information, further research is needed to improve its transparency and integration with personalized health data to be a more usable tool in making informed health decisions.

## Introduction

### Background: Recurrent Urinary Tract Infections for Individuals With Spinal Cord Injury or Disease

Each year, urinary tract infections (UTIs) impact approximately 150 million people worldwide [1]. UTIs represent a substantial burden on health care systems due to frequent emergency department visits and the complexities of managing recurrent cases. Among individuals with spinal cord injury or disease (SCI/D), including multiple sclerosis and spina bifida, the burden of UTIs is more pronounced, with nearly 60% experiencing recurrent UTIs each year—a rate 30 times higher than that of the general population [2,3]. Recurrent UTIs are the leading cause of hospital readmissions for individuals with SCI/D, underscoring the challenges in both symptom management and timely diagnosis [4,5].

### Challenges in Symptom Recognition and Treatment

The disproportionately high prevalence of UTIs in SCI/D populations is linked to neurogenic bladder dysfunction, a condition often requiring catheterization—either intermittent or indwelling—that significantly increases susceptibility to bacterial colonization and infection [6,7]. Unlike the general population, individuals with SCI/D frequently exhibit atypical UTI symptoms such as fatigue, cloudy or malodorous urine, and abdominal discomfort [8,9]. The atypical symptomatology complicates UTI recognition and delays diagnosis, contributing to further health risks and reduced quality of life [10,11].

This combination of persistent bacteria in the urine and atypical infection symptoms makes it challenging to determine when antibiotic treatment is needed, potentially leading to both delayed treatment of serious infections and unnecessary antibiotic use for benign bacteriuria. Misdiagnoses often lead to unnecessary antibiotic prescriptions, increasing the risk of antimicrobial resistance and placing additional strain on the health care system [12,13]. Addressing these symptom presentation challenges requires reliable, standardized tools that empower individuals with SCI/D to accurately identify and manage their symptoms.

### Limitations in the Use of Validated Symptom Assessment Tools

The Urinary Symptom Questionnaires for Neurogenic Bladder (USQNB) is a validated set of instruments designed to capture nuanced symptom profiles across different bladder management methods, including intermittent catheterization, indwelling catheters, and independent voiding [14-17]. While the USQNB tool demonstrates strong alignment with patient-reported outcomes and provides a robust framework for clinical use, its application remains inconsistent in practice [5,15]. Many individuals with SCI/D instead rely on less reliable resources such as general internet searches or anecdotal advice, which can lack accuracy and relevance [18,19].

Validated tools like the USQNB exist to support symptom assessment, yet their limited adoption highlights the need for more accessible solutions that can provide immediate personalized guidance. Despite the availability of validated tools, like the USQNB, individuals with SCI/D often turn to general internet searches or anecdotal advice for symptom assessment. Digital platforms (eg, internet) are an increasingly common source of health information to support symptom assessment, with over 70% of users reporting online searches for health-related content [20,21]. Digital platforms offer accessibility and immediacy but are criticized for propagating misinformation, leading to anxiety, ineffective self-management, and delays in seeking professional care [22,23]. ChatGPT, along with other large language models, has been increasingly integrated into health information seeking and chronic care contexts, offering decision-making support and patient engagement across a range of conditions [24-26].

### Opportunities and Risks of Digital Tools

ChatGPT has demonstrated potential utility in health care settings, including clinical decision support, patient education, and symptom interpretation [27,28]. Recent qualitative work shows that patients are already using ChatGPT to interpret symptoms and guide care-seeking behavior while also raising concerns about reproducibility and source credibility [29]. While early studies have shown promising results in general medical applications, ChatGPT’s effectiveness for specific populations with complex health care needs, such as individuals with SCI/D, remains unexplored. ChatGPT’s ability to process natural language and provide contextual responses could be especially valuable for individuals with SCI/D who often face challenges in interpreting their unique symptoms.

### The Role of ChatGPT in SCI/D Symptom Management

For individuals with SCI/D, ChatGPT’s ability to provide immediate tailored responses could help address barriers to timely health care access, such as mobility limitations and the need for specialized expertise [12,13]. However, ChatGPT’s usefulness depends on patients’ perceptions of accuracy and relevance, as well as its integration with existing health care practices. Other studies have shown that chatbot-based tools are acceptable and potentially beneficial for individuals with complex or stigmatized conditions, including those with limited access to traditional care [30]. Recent work has also examined generative artificial intelligence (AI) errors in health care settings, including a preliminary classification system that categorized errors by input and output stages, identifying omission errors as the most common in both patient-facing and clinical documentation large language model applications [31]. Understanding how individuals with SCI/D perceive and use ChatGPT is critical for evaluating its potential as a reliable tool for managing urinary symptoms.

This study aims to explore the use and perceptions of ChatGPT for urinary symptom management among individuals with SCI/D, addressing the following objectives:

- Identify the types of urinary symptom–related information users seek through ChatGPT
- Evaluate the perceived accuracy, usefulness, and limitations of ChatGPT’s responses
- Examine how ChatGPT-derived information is integrated with existing health resources and professional medical recommendations, using valid and reliable patient-centered, patient-reported outcomes (USQNB)
- Assess the impact of ChatGPT use on self-management practices and health care engagement

By addressing these objectives, this research seeks to advance the understanding of AI’s role in supporting individuals with complex health care needs.

## Methods

### Overview

The 2-phase study approach examined the use and perceptions of ChatGPT for urinary symptom management among individuals with SCI/D.

- Phase 1 consisted of scenario-based testing in which participants interacted with ChatGPT (version 3.5) using scenarios based on validated patient-reported outcome tools, specifically the USQNB for intermittent and indwelling catheter users.
- Phase 2 consisted of follow-up interviews to assess participants’ experiences using ChatGPT for urinary symptom management advice.

### Ethical Considerations

The MedStar Health Research Institute Institutional Review Board approved this study (IRB #00007397). Participants received a US $50 check as compensation following completion of the interview.

### Interview Guide Creation

The semistructured interview guide was developed using the two USQNB instruments for intermittent and indwelling catheter users, both are validated instruments with established reliability [15-17]. The USQNB comprises distinct instruments for different bladder management methods (intermittent catheters, indwelling catheters, and independent voiding, which was not included in this study) specific to individuals with neurogenic lower urinary tract dysfunction. These tools were adapted to capture a range of patient-reported experiences. The scenario-based testing involved:

- providing demographics, health information–seeking behaviors, and technology comfort level;
- sharing experiences with urinary symptoms and UTIs, including specific instances of seeking or considering medical attention for a possible UTI;
- interacting with ChatGPT for urinary symptom management scenarios; and
- completing a follow-up survey on trust and agreement with ChatGPT.

The interview guide included questions on participants’ agreement with ChatGPT’s symptom management recommendations, comfort level using AI technology for health care decisions, perceived limitations and concerns, typical approaches to health care decision-making, and preferences for receiving medical advice.

### Data Collection

Participants were purposively sampled from advocacy groups, social media platforms, and the MedStar National Rehabilitation Hospital outpatient network [32]. The National Rehabilitation Hospital serves over 400,000 patients annually and maintains a database of more than 300 individuals with spinal cord injury or neurogenic bladder dysfunction. Participants were selected based on the following inclusion criteria:

- Diagnosed with SCI, multiple sclerosis, or spina bifida
- Experienced recurrent urinary symptoms requiring management (intermittent or indwelling catheter)
- Ability and willingness to participate in remote scenario-based testing

Eligible participants were contacted through direct outreach, leveraging partnerships with advocacy organizations and clinical referrals. Recruitment materials emphasized the minimal risks involved and highlighted the importance of participant contributions to advancing research on AI-driven health care solutions.

Participants interacted with ChatGPT using scenarios that simulated real-world symptom management challenges for individuals with SCI/D. These scenarios were based on validated patient-reported outcome tools for bladder management, specifically the USQNB [15-17]. Follow-up interviews were conducted to capture participants’ perceptions of ChatGPT’s usability, accuracy, and value in managing their symptoms. After the interviews, participants completed a 5-item survey that included rating their level of agreement with ChatGPT’s responses.

The scenario-based testing was conducted remotely using Microsoft Teams (version 2406), which expanded the geographic reach and provided flexibility by eliminating commute time. Before testing began, each participant provided verbal consent. Follow-up interviews lasted approximately 30-60 minutes and were recorded for transcription and analysis. The average interview duration was 34.0 (SD 15.9, range 8-67) minutes, for a total of 17 hours and 9 minutes. The interviewer (AKR) encouraged participants to elaborate on their experiences, particularly focusing on their interactions with ChatGPT and how it influenced their symptom management decisions.

### Data Analysis

Interview transcripts were cleaned and deidentified. Two researchers (IN, DNB) organized the data in Microsoft Excel (version 2406), with columns for each interview question and respective responses from all 30 interviewees.

The research team conducted an inductive content analysis [33] of the interview data. Two researchers (BZH, AKR) developed a codebook iteratively with four emerging themes: experiences with ChatGPT, decision-making influence, health and technology use, and symptom management.

Three researchers (BZH, IN, DNB) independently coded interview questions for each of the four main codes. Researchers met periodically and resolved discrepancies through consensus-based discussions. During discussions, the team iteratively refined code definitions and identified emerging subthemes.

To ensure rigor, the team used peer debriefing and maintained an audit trail of research decisions [32,34]. The final analysis involved identifying patterns within and across themes, synthesizing findings to address our research questions about experiences using ChatGPT for urinary symptom management advice and its influence on health care engagement decisions.

## Results

### Participants

A total of 30 individuals with SCI/D participated in this study. Table 1 summarizes participant characteristics, including demographics and clinical details.

**Table 1.** Study participant characteristics.^a^

	Participants (N=30)
Gender, n (%)	
Male	20 (67)
Female	10 (33)
Age (years)	
Range	31-75
Mean (SD)	53.9 (14.0)
Race/ethnicity, n (%)	
White	27 (90)
Black or African American	2 (7)
Asian	1 (3)
Location, n (%)	
Suburban	20 (67)
Urban	7 (23)
Rural	3 (10)
Primary conditions, n (%)	
Spinal cord injury	20 (67)
Multiple sclerosis	8 (27)
Spina bifida	2 (7)
Catheter use, n (%)	
Indwelling catheter	8 (27)
Intermittent catheter	22 (73)
Annual household income (US $), n (%)	
≥$150,000	11 (37)
$100,000-$149,999	2 (7)
$75,000-$99,999	3 (10)
$50,000-$74,999	8 (27)
$25,000-$49,999	4 (13)
<$25,000	2 (7)
Technology use, n (%)	
Comfortable or extremely comfortable	29 (97)
Medium comfortability	1 (3)
Technologies used, n (%)^b^	
Google	22 (73)
Amazon Alexa	10 (33)
Siri	5 (17)
ChatGPT	4 (13)

a The percentages do not add up to 100 in some categories due to rounding.

b Participants could select multiple options for this question.

### Themes

#### Overview

Our team identified four emerging themes:

- Symptom management: how participants experience, interpret, and respond to urinary symptoms in their daily lives, including their physical manifestations, functional impacts, and coping strategies
- Experiences with ChatGPT: how participants evaluate and perceive ChatGPT’s reliability, capabilities, and limitations for urinary symptom management, including their assessment of its accuracy and comparisons to other information sources
- Decision-making influence: how participants use ChatGPT to inform choices about symptom assessment, self-management strategies, and timing of professional medical care engagement
- Health and technology use: how participants typically access health information and engage with technology in their daily lives, including their comfort level with digital tools and preferred sources for medical advice

#### Symptom Management

Table 2 shows three key management considerations: daily life impacts, symptom characteristics, and management approaches. A total of 5 participants reported fatigue, 6 reported pain, and 4 reported having a fever; 5 noted emotional impacts, and 3 described social limitations. Among 29 participants discussing symptoms, 7 reported cloudy urine, 5 reported malodorous urine, and 5 reported dark urine. A total of 6 participants experienced increased frequency, and 4 reported leakages. Of 28 participants describing management strategies, 15 increased water intake and 18 used supplements. A total of 5 participants used medications, and 4 described equipment management techniques.

**Table 2.** Thematic analysis of ChatGPT use by individuals with spinal cord injury or disease (SCI/D) for urinary symptom management.

Theme and component		Definition	Exemplary quote
Urinary symptom management			
Daily life impact		How urinary symptoms affect daily activities	“It depends on the severity; catheter clogging can prevent me from leaving the house, discomfort and pain, and restriction on going out in public.” (P20) ”Sickness, nausea, smelly urine, affects how I interact with people often - the main issue is I try not to get close to people due to smelly urine.” (P26)
Symptom characteristics		Frequently experienced urinary symptoms	“If these symptoms occur individually: abdominal pain, dizziness, decreased urine volume, worse smelling volume, darker urine, increase in fatigue, loss of appetite, feeling unwell, altered sleeping patterns, muscle aches, increased positional pain, nausea, change in bowel patterns, abdominal discomfort or bloating, irritability or mental slowing. If multiple occur together, I may call the doctor. I typically wait until it gets bad.” (P18)
Management approaches		Methods used for self-management	“I’ve taken everything from cranberry pills to...garlic pills. I’ve read all these different things where people find certain diets to help less. Sugars, less starches, cause starches turn into sugars, more proteins, increased fluid intake...I’ve tried all of the above.” (P7)
Experiences with ChatGPT			
Information accuracy assessment		How participants evaluate ChatGPT’s reliability and alignment with professional medical guidance	“I don’t think it was the best…It could be more beneficial if it knew more about my medical record…if it could connect with my chart.” (P22)
Information source comparison		How participants compare ChatGPT to other health information sources	“Yes, as long it’s correct and there is some background that it’s coming from a reliable source” (P25) “First I would use my web portals, because that’s trained doctors and nurses” (P18)
Decision-making influence			
Symptom evaluation		How participants assess specific health symptoms and their severity	“I have muscle aches, I feel unwell, and I have abdominal discomfort” (P3) “ChatGPT, I’m experiencing cloudiness, dark color, urine leakage, and increased odor in my urine. Should I seek medical advice?” (P14)
Guidance seeking		How participants use ChatGPT’s recommendations for health care decisions	“How long should these symptoms persist before I seek treatment: back pain, fever, abdominal bloating, overall fatigue, increased frequency, urgency, odor, and cloudiness?” (P6) “The incontinence Is not excessive, but more than usual. It doesn’t smell and is cloudy each time I cath. Are there certain solutions I can try myself before I seek antibiotics?” (P27)
Patterns in health information seeking and technology use			
Health information sourcing		Primary methods for obtaining health advice	“I go to my general practitioner or research on Web MD-type places.” (P12) “Doctors, PubMed, family and friends that are physicians” (P20)
Technology engagement		Level of technology use for health information	“Comfortable but not 100% with technology.” (P29)

#### Experiences With ChatGPT

Table 2 presents two perspectives on information evaluation: accuracy assessment and source comparison. Of the 30 participants, 13 strongly agreed and 17 agreed with ChatGPT’s advice, and 5 participants noted alignment with professional medical guidance. For example, one participant shared, “It seems to be on target with what the professionals tell me.” ChatGPT provided suggestions such as “monitor symptoms for 48 hours before seeking care” or “increase fluid intake to alleviate mild urinary symptoms,” which participants often described as clear and actionable.

Some participants, however, indicated that ChatGPT’s advice lacked the specificity they needed. Suggestions like “Seek medical advice if symptoms persist beyond 72 hours” were perceived as too general, particularly when participants expected advice tailored to their individual health histories.

#### Decision-Making Influence

Table 2 shows two health care engagement patterns: symptom evaluation and care guidance. A total of 29 participants described ChatGPT’s influence on health care decisions, 17 assessed their urine characteristics, 6 inquired about pain, and 5 noted urination pattern changes. One participant exemplified typical queries: “I’m experiencing cloudiness, dark color, urine leakage, and increased odor in my urine, should I seek medical advice?”

Among the 28 participants seeking care guidance, 12 used ChatGPT to determine when to seek professional help, 9 sought self-management guidance, and 7 requested urgent care advice.

#### Health and Technology Use

Table 2 presents two information access patterns: health information sourcing and technology engagement. Of the 30 participants, 27 consulted doctors for health advice, with 5 mentioning specialists specifically. A total of 15 used internet resources, and 4 consulted family members with medical backgrounds. Of the 30 participants, 29 reported regular technology use. Most described high comfort levels, though age-related challenges were noted; for instance, one participant explained, “[Th]is a learning experience for an older guy.”

## Discussion

### Patient Trust and the Promise of Generative AI

This study provides novel insights into how individuals with SCI/D perceive and use ChatGPT (version 3.5) for managing urinary symptoms. Our findings demonstrate both promising applications and important limitations of AI-driven health information tools in supporting chronic condition management. These findings align with a recent generative AI study reporting that 42% of errors in patient-facing language models were omissions, with 25% of the errors having high clinical significance [31]. These results also echo prior studies highlighting ChatGPT’s potential in chronic care, mental health, and patient education while also raising concerns about reproducibility and trustworthiness [26,30]. The high level of trust in ChatGPT reported by participants is consistent with emerging literature on AI acceptance in health care settings [27]. However, this trust must be considered within the broader challenges faced by individuals with SCI/D in interpreting urinary symptoms, especially given the atypical presentation of UTIs and difficulties in symptom interpretation highlighted by previous research [8,9].

### ChatGPT’s Role in Supporting Symptom Interpretation

Our findings regarding symptom assessment patterns are particularly noteworthy when considered alongside existing literature on UTI management in SCI/D populations. Participants frequently sought guidance on assessing urine characteristics, pain, and urination pattern changes—symptoms that often present atypically in this population [3]. These behaviors align with other qualitative studies in which patients used ChatGPT to make sense of ambiguous symptoms and to guide decisions about care seeking [29]. These findings suggest ChatGPT may serve as a valuable tool in helping individuals interpret their symptoms, potentially addressing the gap between symptom onset and clinical intervention that has been identified as a significant challenge in UTI management [4,5]

The role of ChatGPT in health care decision-making emerged as a key theme, with 28 participants using it to guide care-seeking behaviors. This finding is significant given the high rates of emergency department visits and hospital readmissions for UTIs among individuals with SCI/D [2]. ChatGPT’s ability to provide immediate, tailored feedback may help address the uncertainty in distinguishing between asymptomatic bacteriuria and true UTIs—a distinction that has proven challenging even in clinical settings [35].

### Limitations of ChatGPT in Personalized Care

However, participants’ concerns about ChatGPT’s lack of integration with personal health records echo broader challenges in health information technology adoption. This limitation is particularly relevant given the importance of standardized assessment tools, like the USQNB [14], and the need for contextualized symptom evaluation in neurogenic bladder management. Future development of AI-driven health tools should prioritize integration with validated assessment instruments and personal health data to enhance their utility in chronic condition management.

### Study Limitations and Opportunities for Future AI-Driven Care

The study revealed that while participants maintain strong relationships with health care providers, they value ChatGPT as a complementary resource. This mirrors findings from studies in perinatal mental health, where patients describe chatbots as supportive and scalable companions between formal care encounters [30]. Such tools may help bridge the gap between professional care visits, potentially reducing delays in symptom recognition and treatment initiation that have been associated with poorer outcomes in this population [12].

There are some study limitations. First, our sample was limited to individuals with SCI/D who had extensive experience managing urinary symptoms and were predominantly White (27/30) and technologically proficient (29/30 comfortable with technology). Despite recruitment efforts through multiple platforms, including advocacy groups, social media, and clinical networks, the study sample lacked racial and ethnic diversity. Thus, findings may not be generalizable to newly diagnosed patients or those with less experience in symptom management. Additionally, while the remote scenario-based testing format enhanced accessibility, it limited our ability to observe contextual factors influencing real-world use. Future research should prioritize the recruitment of racially, ethnically, and socioeconomically diverse participants, including individuals newly navigating urinary symptom management. In addition, future research should examine how AI tools, like ChatGPT, can be better integrated with validated condition-specific instruments such as the USQNB to enhance clinical relevance and accuracy. Longitudinal studies are also needed to assess whether AI use improves symptom recognition, self-management behaviors, care-seeking decisions, and clinical outcomes over time. These directions will help clarify the role of generative AI in improving access, quality, and safety of care for individuals with SCI/D and other populations managing chronic conditions.

This study provides important insights into the potential role of AI chatbots like ChatGPT in supporting urinary symptom management for individuals with SCI/D. While participants found value in using ChatGPT to verify their knowledge and potentially gain additional information, it is clear that these tools should complement rather than replace existing health management strategies and professional medical care. As AI continues to evolve, careful consideration must be given to how it can be optimally integrated into chronic disease management to enhance patient care while maintaining appropriate safeguards and supporting established clinical relationships.

### Conclusion

Individuals with SCI/D generally find ChatGPT to be a trustworthy and valuable tool for managing urinary symptoms while still relying on their personal experience and professional medical advice for decision-making. As AI technology continues to advance, it has the potential to serve as a complementary tool in chronic condition management. Generative AI tools should consider attention to personalization, integration with existing health care systems, and ethical considerations to be highly usable for individuals managing chronic conditions like SCI/D.

## Abbreviations

AI: artificial intelligence
SCI/D: spinal cord injury or disease
USQNB: Urinary Symptom Questionnaires for Neurogenic Bladder
UTI: urinary tract infection

## References

[1] Stamm WE, Norrby SR (2001). Urinary tract infections: disease panorama and challenges. J Infect Dis.

[2] Garcia-Arguello LY, O’Horo JC, Farrell A (2017). Infections in the spinal cord-injured population: a systematic review. Spinal Cord.

[3] Skelton-Dudley F, Doan J, Suda K, Holmes SA, Evans C, Trautner B (2019). Spinal cord injury creates unique challenges in diagnosis and management of catheter-associated urinary tract infection. Top Spinal Cord Inj Rehabil.

[4] Craven BC, Alavinia SM, Gajewski JB (2019). Conception and development of urinary tract infection indicators to advance the quality of spinal cord injury rehabilitation: SCI-High Project. J Spinal Cord Med.

[5] Groah SL, Ljungberg IH, Tractenberg RE, Rounds AK (2020). Self-Management of Urinary Symptoms Using a Probiotic in People with Spinal Cord Injuries, Spina Bifida, and Multiple Sclerosis.

[6] Dahlberg A, Perttilä I, Wuokko E, Ala-Opas M (2004). Bladder management in persons with spinal cord lesion. Spinal Cord.

[7] Salameh A, Mohajer MA, Daroucihe RO (2015). Prevention of urinary tract infections in patients with spinal cord injury. CMAJ.

[8] Linsenmeyer TA, Oakley A (2003). Accuracy of individuals with spinal cord injury at predicting urinary tract infections based on their symptoms. J Spinal Cord Med.

[9] Wheeler TL, de Groat W, Bowel and Bladder Workshop Participants (2018). Translating promising strategies for bowel and bladder management in spinal cord injury. Exp Neurol.

[10] Hooton TM, Bradley SF, Cardenas DD (2010). Diagnosis, prevention, and treatment of catheter-associated urinary tract infection in adults: 2009 International Clinical Practice Guidelines from the Infectious Diseases Society of America. Clin Infect Dis.

[11] Pannek J (2011). Treatment of urinary tract infection in persons with spinal cord injury: guidelines, evidence, and clinical practice. J Spinal Cord Med.

[12] Madden-Fuentes RJ, McNamara ER, Lloyd JC (2013). Variation in definitions of urinary tract infections in spina bifida patients: a systematic review. Pediatrics.

[13] Stillman MD, Hoffman JM, Barber JK, Williams SR, Burns SP (2018). Urinary tract infections and bladder management over the first year after discharge from inpatient rehabilitation. Spinal Cord Ser Cases.

[14] Rounds AK, Tractenberg RE, Groah SL (2023). Urinary symptoms are unrelated to leukocyte esterase and nitrite among indwelling catheter users. Top Spinal Cord Inj Rehabil.

[15] Tractenberg RE, Groah SL, Rounds AK, Ljungberg IH, Schladen MM (2018). Preliminary validation of a Urinary Symptom Questionnaire for individuals with Neuropathic Bladder using Intermittent Catheterization (USQNB-IC): a patient-centered patient reported outcome. PLoS One.

[16] Tractenberg RE, Frost JK, Yumoto F, Rounds AK, Ljungberg IH, Groah SL (2021). Reliability of the Urinary Symptom Questionnaires for people with neurogenic bladder (USQNB) who void or use indwelling catheters. Spinal Cord.

[17] Tractenberg RE, Frost JK, Yumoto F, Rounds AK, Ljungberg IH, Groah SL (2021). Validity of the Urinary Symptom Questionnaires for people with neurogenic bladder (USQNB) who void or use indwelling catheters. Spinal Cord.

[18] Hearn JH, Selvarajah S, Kennedy P, Taylor J (2018). Stigma and self-management: an interpretative phenomenological analysis of the impact of chronic recurrent urinary tract infections after spinal cord injury. Spinal Cord Ser Cases.

[19] Mays R, McIntyre A, Mehta S, Hill D, Wolfe D, Teasell R (2014). A review of educational programs to reduce UTIs among individuals with SCI. Rehabil Nurs.

[20] Fox S (2014). The social life of health information. Pew Research Center.

[21] Prestin A, Vieux SN, Chou WYS (2015). Is online health activity alive and well or flatlining? Findings from 10 years of the Health Information National Trends Survey. J Health Commun.

[22] Tan SSL, Goonawardene N (2017). Internet health information seeking and the patient-physician relationship: a systematic review. J Med Internet Res.

[23] Wang J, Ashvetiya T, Quaye E, Parakh K, Martin SS (2018). Online health searches and their perceived effects on patients and patient-clinician relationships: asystematic review. Am J Med.

[24] Garg RK, Urs VL, Agarwal AA, Chaudhary SK, Paliwal V, Kar SK (2023). Exploring the role of ChatGPT in patient care (diagnosis and treatment) and medical research: a systematic review. Health Promot Perspect.

[25] Ray PP (2023). ChatGPT: a comprehensive review on background, applications, key challenges, bias, ethics, limitations and future scope. Internet Things Cyber Phys Syst.

[26] Alhur A (2024). Redefining healthcare with artificial intelligence (AI): the contributions of ChatGPT, Gemini, and Co-pilot. Cureus.

[27] Harskamp RE, De Clercq L (2024). Performance of ChatGPT as an AI-assisted decision support tool in medicine: a proof-of-concept study for interpreting symptoms and management of common cardiac conditions (AMSTELHEART-2). Acta Cardiol.

[28] Nedbal C, Naik N, Castellani D, Gauhar V, Geraghty R, Somani BK (2024). ChatGPT in urology practice: revolutionizing efficiency and patient care with generative artificial intelligence. Curr Opin Urol.

[29] Schütz P, Lob S, Chahed H (2024). ChatGPT as an information source for patients with migraines: a qualitative case study. Healthcare (Basel).

[30] McAlister K, Baez L, Huberty J, Kerppola M (2025). Chatbot to support the mental health needs of pregnant and postpartum women (Moment for Parents): design and pilot study. JMIR Form Res.

[31] Hose BZ, Handley JL, Biro J (2025). Development of a preliminary patient safety classification system for generative AI. BMJ Qual Saf.

[32] Devers KJ (1999). How will we know “good” qualitative research when we see it? Beginning the dialogue in health services research. Health Serv Res.

[33] Elo S, Kyngäs H (2008). The qualitative content analysis process. J Adv Nurs.

[34] Robson C, McCartan K (2016). Real World Research.

[35] Gupta K, Hooton TM, Naber KG (2011). International clinical practice guidelines for the treatment of acute uncomplicated cystitis and pyelonephritis in women: a 2010 update by the Infectious Diseases Society of America and the European Society for Microbiology and Infectious Diseases. Clin Infect Dis.

